# Molecular dynamics study of water anomalies: a comparison of OPC3 and TIP4P/$\varepsilon$ models

**DOI:** 10.1007/s00894-026-06748-x

**Published:** 2026-05-15

**Authors:** Vanderson dos S. Nascimento, Rogelma M. S. Ferreira, Marcia C. Barbosa, José Rafael Bordin

**Affiliations:** 1 Programa de Pós-Graduação em Física, Instituto de Física, Universidade Federal do Rio Grande do Sul, Porto Alegre, RS Brazil; 2 Centro de Ciências Exatas e Tecnológicas, Universidade Federal do Recôncavo da Bahia, Cruz das Almas, BA Brazil; 3 Instituto de Física, Universidade Federal do Rio Grande do Sul, Porto Alegre, RS Brazil; 4 Departamento de Física, Instituto de Física e Matemática, Universidade Federal de Pelotas, Pelotas, RS Brazil; 5 Fachbereich Physik, Universität Konstanz, Konstanz, Deutschland

**Keywords:** Water models, Rigid non-polarizable force fields, Water anomalies, Molecular dynamics simulations

## Abstract

**Context:**

Classical water models are commonly parameterized to reproduce selected experimental properties, with the dielectric constant often treated as a primary target. However, improving dielectric response within rigid, non-polarizable frameworks can redistribute errors and affect the simultaneous description of structural, thermodynamic, and dynamic properties. In this work, we investigate this trade-off by comparing two widely used rigid water models with distinct parameterization strategies: the four-site TIP4P/$\varepsilon$ model, designed to enhance dielectric properties relative to TIP4P/2005, and the three-site OPC3 model, optimized for overall thermodynamic and dynamic performance. By analyzing structural correlations, density and diffusion anomalies, and excess entropy, we show that both models reproduce the qualitative hierarchy of water-like anomalies, while exhibiting systematic quantitative differences linked to their molecular architecture. TIP4P/$\varepsilon$ provides a more accurate description of short-range structure and density-related anomalies, whereas OPC3 displays softer intermediate-range correlations with comparable dynamic behavior. These results demonstrate that anomaly-based analyses offer a sensitive framework for assessing the redistribution of accuracy induced by different force-field parameterization choices and confirm the continued relevance of rigid water models for large-scale simulations of liquid water.

**Methods:**

Molecular dynamics simulations were performed using the LAMMPS package. Liquid water was modeled using the rigid, non-polarizable OPC3 and TIP4P/$\varepsilon$ force fields, combining Lennard–Jones and Coulombic interactions. Simulations were carried out over broad ranges of temperature and density using Nosé–Hoover thermostat and barostat schemes. Structural properties were analyzed through radial distribution functions, thermodynamic anomalies were assessed from density–temperature relations, and molecular mobility was quantified via diffusion coefficients. Excess entropy was estimated from pair correlation functions to rationalize the coupling between structure and dynamics.

**Supplementary Information:**

The online version contains supplementary material available at 10.1007/s00894-026-06748-x.

## Introduction

Fluids are fundamental to life across macroscopic and microscopic scales, and among them water occupies a unique position due to its central role in biological, environmental, and technological processes. Beyond its societal importance, water is also a paradigmatic system in molecular modeling, as it exhibits thermodynamic and dynamic anomalies that sharply distinguish it from simple liquids [1]. These include the temperature of maximum density, anomalous compressibility, and non-monotonic diffusion behavior, which make water a stringent benchmark for interaction potentials and simulation methodologies [2–4].

At the microscopic level, such anomalies originate from the competition between distinct local structural arrangements mediated by hydrogen bonding [5, 6], leading to a strong coupling between structure, thermodynamics, and molecular mobility. This coupling has been extensively characterized through experimental scattering techniques, providing essential reference data for validating molecular models [7–11]. Anomalous behavior is also relevant for water transport under confinement, motivating studies on filtration and desalination in nanoporous materials [12–15].

Over the past decades, a broad hierarchy of classical water models has been developed, ranging from rigid non-polarizable force fields to flexible, polarizable, and more recently data-driven formulations. Early rigid models such as TIP3P, TIP4P, SPC, and SPC/E established the foundations of molecular simulations of water [16–18], while subsequent refinements increased the number of interaction sites or optimized parameterization strategies to improve agreement with experimental structural and thermodynamic properties [19–23]. Flexible models explicitly incorporating intramolecular degrees of freedom were later introduced, offering improved realism in specific thermodynamic regimes [24, 25].

Polarizable models, based on induced dipoles or Drude oscillators, further increased physical realism and improved dielectric and solvation properties, albeit at a substantially higher computational cost [26–30]. More recently, many-body and machine-learning-based approaches have achieved near quantum-level accuracy [31], as exemplified by the MB-pol potential [32, 33] and by data-driven models such as Gaussian Approximation Potentials and neural network potentials [34–36]. Applications combining neural-network-based molecular dynamics with active learning strategies further demonstrate the ability of these models to reproduce structural, thermodynamic, and dynamic properties of water across broad thermodynamic conditions [37]. Despite these advances, their computational overhead, training complexity, and limited transferability still pose challenges for extensive sampling, long timescale simulations, and systematic benchmarking studies.

For these reasons, rigid non-polarizable models remain widely employed in molecular dynamics simulations of water [38]. In applications focusing on phase behavior, equations of state, and transport properties in the absence of strong external electric fields, such models offer a robust and numerically stable description governed solely by the potential energy surface (PES), enabling extensive sampling at a favorable accuracy-to-cost ratio [2, 39]. Their global performance can be systematically assessed: the benchmarking strategy proposed by Vega and Abascal shows that some four-site models, notably TIP4P/2005, achieve high overall scores across a broad set of properties, even though no rigid non-polarizable model reproduces all experimental observables simultaneously [2, 40]. Recent work further indicates that non-polarizable four-site models are approaching a practical accuracy limit; in particular, enforcing agreement with the experimental dielectric constant may redistribute errors rather than improve the overall description of thermodynamic, phase-equilibrium, and transport properties, since the dielectric response depends not only on the PES but also on the dipole-moment surface [40, 41].

The continued success of the Madrid family of force fields—combining rigid TIP4P/2005 water with scaled ionic charges—demonstrates that such models can nevertheless provide accurate and computationally efficient descriptions of aqueous solutions over wide concentration ranges [42]. Large-scale benchmarking studies therefore confirm that different rigid models offer distinct compromises between structural fidelity, thermodynamic accuracy, and dynamical behavior [3, 22, 39]. Building on this established framework, modern rigid three-site and four-site water models continue to attract attention as practical and well-controlled representations of the water PES. The TIP4P/$\varepsilon$ model can be viewed as a deliberate modification of the TIP4P/2005 force field, designed to enhance the dielectric response while preserving the underlying four-site geometry and interaction topology [43]. The TIP4P family strongly relies on selecting the model parameters on the basis of thermodynamic properties.

In parallel, the OPC3 model represents a state-of-the-art three-site formulation optimized to provide a balanced description of thermodynamic and dynamic properties at reduced computational cost [44]. While offering fewer degrees of freedom to be parameterized compared with TIP4P models, it adopts a strategy that aims to capture both dynamic and thermodynamic properties. This strategy eliminates the constraints on charge values and positions commonly employed in three-site models, optimizing the charge distribution to reproduce the dipole and quadrupole moments of water. This is achieved through a search for fixed-point charge model parameters within the low-dimensional electrostatic subspace of multipole moments.

Rather than performing a global force-field ranking in the spirit of Vega–Abascal benchmarks [2], the present work adopts a complementary perspective by focusing on water-like anomalies as sensitive probes of the coupling between structure, thermodynamics, and dynamics, and how they are affected by the two distinct strategies represented by the two models. Our perspective is in line with the ideas that structural order is related to the anomalies [45, 46], which demonstrated that water anomalies exhibit a hierarchy in which the structural anomalous behavior occurs at a larger region in the pressure temperature phase diagram, being a precursor of the dynamic and thermodynamic anomalies.

In this direction, we perform molecular dynamics simulations of liquid water using the OPC3 and TIP4P/$\varepsilon$ models and compare their predictions with experimental data. Structural properties are analyzed via radial distribution functions, thermodynamic anomalies are assessed through the temperature dependence of density, and molecular mobility is characterized by diffusion coefficients. Excess entropy is employed as a complementary descriptor to rationalize the coupling between microscopic structure and macroscopic transport, providing additional insight into anomalous behavior and into the relative performance of the two models. This systematic comparison elucidates how dielectric-targeted versus globally optimized parameterizations, within three-site and four-site rigid models, redistribute accuracy across thermodynamic, structural, and dynamic anomalies of liquid water.

The remainder of this paper is organized as follows. The “The simulation models and details” section describes the simulation models and computational methodology. The “Results and discussion” section presents and discusses the structural, thermodynamic, and dynamic results, including the analysis of water-like anomalies. The “Conclusions” section summarizes the main conclusions.

## The simulation models and details

The TIP4P/$\varepsilon$ model was introduced by Fuentes-Azcatl and Alejandre in 2014 [43]. It is a four-site model derived from the TIP4P family, specifically reparameterized to improve the dielectric constant while preserving the underlying molecular geometry. This targeted optimization allows the model to reproduce the temperature of maximum density and dielectric response with good accuracy [47]. The OPC3 model was proposed by Izadi and Onufriev in 2016 [48] as a modern three-site formulation. Benchmarking against other widely used three-site models, including mTIP3P, SPC/E, TIP3P-FB [49], and H2ODC [50], demonstrated that OPC3 represents the practical performance limit for rigid, non-polarizable three-site models across a broad temperature range.

OPC3 consists of one oxygen and two hydrogen atoms, whereas TIP4P/$\varepsilon$ includes an additional massless site (M) where the negative charge is located, displaced from the oxygen atom. All models were treated as rigid bodies interacting through effective pair potentials composed of Lennard–Jones and Coulombic terms, with parameters summarized in Table 1 [43, 48]. As the models used are rigid, the intramolecular constraints were enforced using the SHAKE algorithm with a tolerance of 10$^{-4}$. A cutoff radius of 10 Å was used for the interactions, and the P3M method was employed for electrostatic interactions with a tolerance of 10$^{-4}$ [51].

**Table 1:** Parameters for rigid-body water models

Parameter	OPC3	TIP4P/$\varepsilon$
$\sigma$ (Å)	3.17427	3.165
$\varepsilon$ (kcal/mol)	0.163406	0.18481
Charge O (*e*)	−0.89517	–
Charge M (*e*)$^\textrm{a}$	–	−1.054
Charge H (*e*)	0.447585	0.527
Bond O–H (Å)	0.97888	0.9572
Angle H–O–H ($^\circ$)	109.47	104.52
Bond O–M (Å)	–	0.105

Source: [39]

$^\textrm{a}$ In 4-sites models, the oxygen mass center carries its charge, which is displaced to the M site

The simulated system consisted of 500 water molecules under periodic boundary conditions. For density calculations, the simulations were initialized in a cubic box of dimensions 24.64 Å $\times$ 24.64 Å $\times$ 24.64 Å, corresponding to an initial density of 1.0 g/cm$^3$. Each simulation was carried out in three stages: equilibration in the NVT ensemble for 1 ns, followed by pressure equilibration in the NPT ensemble at 100 bar for 1 ns, and a production run of 10 ns in the NPT ensemble at 1 bar. After the equilibration run at 100 bar, the pressure was set to 1 bar, and the system was allowed to re-equilibrate under the new conditions. The initial 2 ns of the 10 ns trajectory were discarded to ensure proper equilibration, and only the remaining portion was used for analysis. Equilibration was assessed by monitoring the time evolution of the system density, total energy, and pressure, which reached a stationary behavior and fluctuated around stable mean values after the transient regime. This procedure was repeated for temperatures ranging from 250 to 350 K in increments of 10 K. The equation of motion was integrated with the velocity-verlet algorithm, with a time step of 1.0 fs, and a Nosé-Hoover thermostat with a damping constant of 10.0 fs was used for temperature balance, and for simulations requiring pressure balance, a barostat with a time constant of 1000.0 fs was used [52–54].

**Fig. 1:**
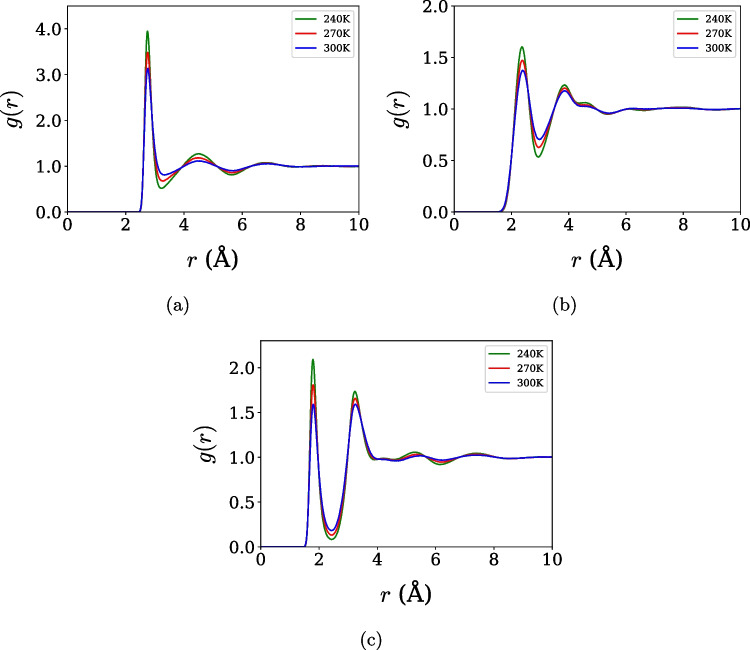
Radial distribution functions for the OPC3 model at three temperatures and with the interactions. (**a**) Oxygen-oxygen, (**b**) hydrogen-hydrogen, and (**c**) oxygen-hydrogen

Diffusion coefficients were obtained from the mean-squared displacement (MSD) using the Einstein relation. For these calculations, the system was equilibrated for 0.6 ns, followed by a 2 ns production run. Diffusion coefficients were obtained from the long-time behavior of the mean-squared displacement using the Einstein relation. The MSD was computed from the Cartesian molecular positions in the laboratory frame, without removing the center-of-mass drift. For each state point, the MSD curve was divided into four time windows within the diffusive regime, linear fits were performed separately in each window, and the final diffusion coefficient was obtained from the average slope. Representative MSD curves and fitting ranges are provided in the Figs. S1 and S2 of the Supplementary Material. Diffusion was evaluated for seven temperatures and eleven distinct densities at each temperature. Finite-size corrections to the diffusion coefficient (e.g., Yeh–Hummer) were not applied, as their effect is known to be small for system sizes of this order and does not affect the qualitative trends discussed here [22].

Excess entropy was employed as a complementary metric to characterize anomalous structural behavior [55]. It can be formally expressed as a sum of two-body and higher-order contributions [3, 56, 57],

1 $$\begin{aligned} S_e = S_2 + S_3 + S_4 + \cdots , \end{aligned}$$

where the two-body contribution is computed from the radial distribution functions (RDFs) as

2 $$\begin{aligned} \frac{S_2}{Nk_B} = -2\pi \rho \int _0^\infty \left[ g(r)\ln g(r) - g(r) + 1\right] r^2 \, dr . \end{aligned}$$

Here, $\rho$ is the number density, *g*(*r*) is the RDF, *N* is the number of particles, and $k_B$ is the Boltzmann constant. Although higher-order contributions are neglected, the pair excess entropy obtained from the O–O, O–H, and H–H radial distribution functions provides a useful qualitative metric to compare structural changes and to rationalize trends in the dynamic anomalies. Deviations from Rosenfeld scaling at low temperatures are interpreted as signatures of the increasing role of many-body correlations beyond the pair level [3, 56]. Moreover, the three-body contribution has been shown to partially cancel higher-order terms [58]. The block averaging method was employed to estimate the density, diffusion coefficient, and excess entropy. Error bars are smaller than the symbol size.

## Results and discussion

**Fig. 2:**
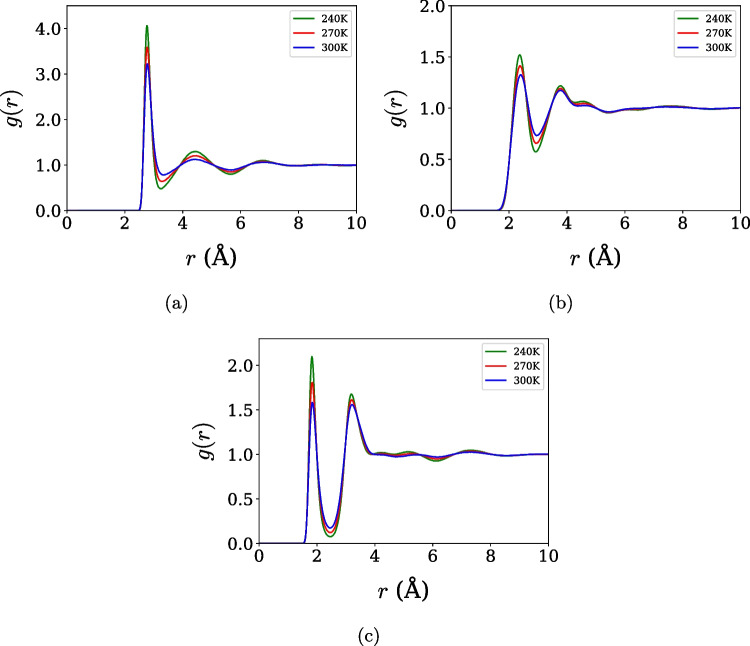
Radial distribution functions for the TIP4P/$\varepsilon$ model at three temperatures and with the interactions. (**a**) Oxygen-oxygen, (**b**) hydrogen-hydrogen, and (**c**) oxygen-hydrogen

The structural properties define how the water molecules are organized, and they are particularly relevant for identifying the water thermodynamic and dynamic anomalous behavior of water [45, 46]. First, the structural properties of liquid water were analyzed through RDFs computed for the OPC3 and TIP4P/$\varepsilon$ models at selected temperatures (240 K, 270 K, and 300 K). Figure 1a, b, and c represent the oxygen-oxygen, hydrogen-hydrogen, and oxygen-hydrogen interactions of the OPC3 model, respectively. Figure 2a, b, and c represent the oxygen-oxygen, hydrogen-hydrogen, and oxygen-hydrogen interaction of the TIP4P/$\varepsilon$ model, respectively. The overall shape and temperature evolution of the RDFs are consistent with the expected response of liquid water, with a gradual reduction of peak intensities and broadening of coordination shells upon heating. These trends indicate a progressive weakening of local structural order and confirm that both models capture the essential features of water structure across the investigated temperature range.

A more quantitative perspective can be obtained from the positions and heights of the first RDF maxima at 300 K, summarized in Table 2 together with experimental data [7]. Both force fields reproduce the locations of the first coordination shells with reasonable accuracy for all atomic pairs, but slightly overestimate the height of the first O–O radial distribution function peak relative to experiment, a behavior commonly observed in rigid, nonpolarizable water models. Such overstructuring reflects the effective nature of the underlying potential energy surface, in which the absence of explicit polarization and intramolecular flexibility leads to sharper short-range correlations. Rather than indicating a failure of the models, this effect is consistent with a known redistribution of accuracy, whereby enhanced short-range order partially compensates for missing many-body effects and enables the reproduction of thermodynamic trends and anomalous behavior. Importantly, despite quantitative deviations in peak heights, the temperature dependence and relative shifts of the RDF maxima remain in good agreement with experiment, supporting the reliability of both models in capturing the structural signatures relevant to water-like anomalies.

**Table 2:** Position ($r$) and intensity value ($g(r)$) of the first maximum of the radial distribution function for the O–O, O–H, and H–H pairs, simulated with the OPC3 and TIP4P/$\varepsilon$ models at 300 K, compared with experimental data

	O–O		O–H		H–H	
	*1*$^{o}$ *maximum*		*1*$^{o}$ *maximum*		*1*$^{o}$ *maximum*	
Model	*r* (Å)	*g*(*r*)	*r* (Å)	*g*(*r*)	*r* (Å)	*g*(*r*)
OPC3	2.757	3.136	1.807	1.589	2.387	1.375
TIP4P/$\varepsilon$	2.785	3.221	1.840	1.583	2.397	1.326
EXP	2.790	2.495	1.860	1.042	2.430	1.339

Souce: Experimental data [7]

**Fig. 3:**
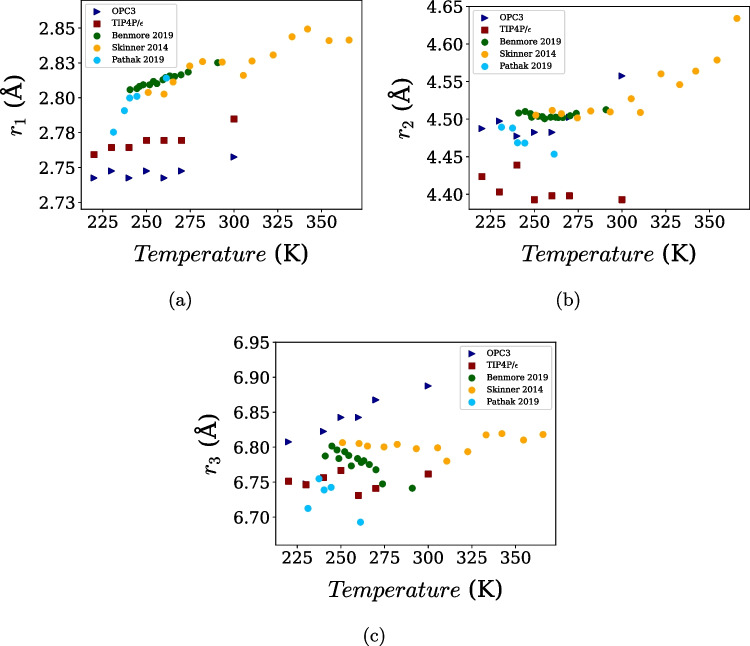
Temperature dependence of the (**a**) first, (**b**) second, and (**c**) third maxima of the O–O radial distribution function for OPC3 and TIP4P/$\varepsilon$, compared with experimental data obtained from the Refs. [8–10]

Further insight into intermediate-range structure is obtained from the temperature dependence of the first three maxima of the O–O RDF, shown in Fig. 3 and compared with experimental scattering data [8–10]. For the two cases, the first (Fig. 3a), second (Fig. 3b), and third (Fig. 3c) coordination shells shift to larger distances with increasing temperature, in qualitative agreement with experiment. Quantitatively, however, systematic differences emerge between the two force fields. TIP4P/$\varepsilon$ shows closer agreement with experimental peak positions for the first and third coordination shells, whereas OPC3 provides a slightly better description of the temperature dependence of the second-shell intensity. This contrast is consistent with the underlying model architectures. Four-site models such as TIP4P/$\varepsilon$ impose stronger angular constraints through the displaced negative charge, leading to a more structured first hydration shell and improved short-range order. In contrast, three-site models like OPC3 tend to soften intermediate-range correlations, which can enhance agreement in the second shell at the expense of reduced accuracy in the first-shell geometry.

These trends are consistent with previous comparative studies. Camisasca et al. [11] reported similar behavior when comparing SPC/E and TIP4P/2005 with MB-pol [32] and experimental data, suggesting that the number of interaction sites plays a central role in determining the accuracy of intermediate-range structural correlations. In this context, the close correspondence between OPC3 and SPC/E, as well as between TIP4P/$\varepsilon$ and TIP4P/2005, reinforces the view that three-site and four-site models form internally consistent families with distinct structural signatures.

**Fig. 4:**
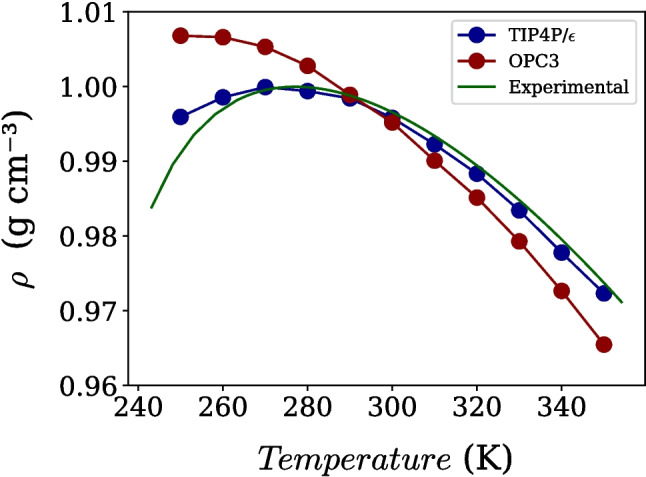
Temperature dependence of water density at 1 bar for the OPC3 and TIP4P/$\varepsilon$ models compared with experimental data [59]

These models were parameterized on the basis of some thermodynamic anomalous behavior. Next, the thermodynamic anomalies were examined by analyzing the density as a function of temperature at 1 bar are shown in Fig. 4. Both models reproduce the characteristic density anomaly of water, displaying a maximum upon cooling. However, clear quantitative differences are observed. TIP4P/$\varepsilon$ reproduces the experimental temperature of maximum density (TMD) at 277 K [59], whereas OPC3 predicts a significantly lower value of approximately 250 K. This difference highlights the sensitivity of density-related anomalies to the balance between electrostatic interactions and excluded-volume effects.

**Fig. 5:**
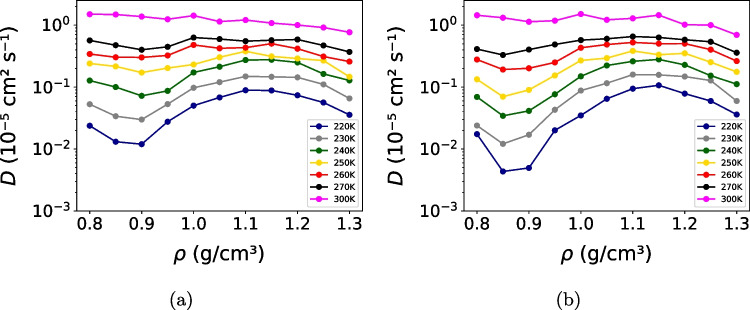
Diffusion coefficient as a function of density for the (**a**) OPC3 and (**b**) TIP4P/$\varepsilon$ models at different temperatures. A non-monotonic dependence of diffusivity on density is observed at low temperatures, characteristic of anomalous transport behavior, which vanishes upon heating. Error bars represent the standard deviation and are smaller than the symbol size

**Fig. 6:**
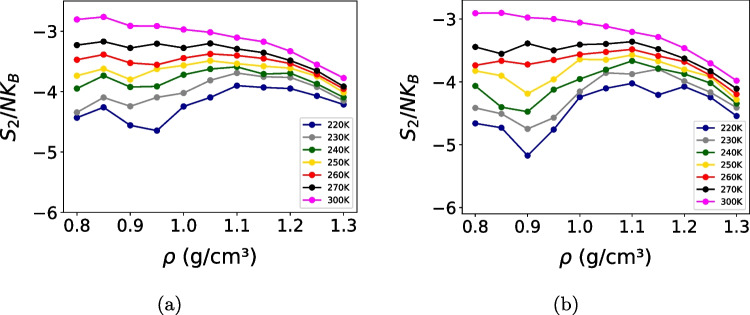
Excess pair entropy as a function of density for the (**a**) OPC3 and (**b**) TIP4P/$\varepsilon$ models at different temperatures. The anomalous region in excess entropy coincides with that of the diffusion coefficient, highlighting the relationship between structural ordering and dynamics. Error bars represent the standard deviation and are smaller than the symbol size

In particular, the improved TMD predicted by TIP4P/$\varepsilon$ is consistent with its reparameterization strategy, which combines an adjusted charge distribution (aimed at a more realistic dielectric response) with a retuned oxygen–oxygen Lennard–Jones interaction. This joint rebalancing of electrostatics and short-range repulsion/dispersion modifies the effective equation of state and the temperature dependence of local structure, yielding a more accurate TMD within the rigid non-polarizable framework.

Another relevant aspect is to balance the thermodynamic and dynamic behavior, which are interlinked in the anomalies of water [45, 46]. Then, the dynamic anomalies were assessed through the diffusion coefficient as a function of density at several temperatures. OPC3 (Fig. 5a) and TIP4P/$\varepsilon$ (Fig. 5b) exhibit the characteristic non-monotonic dependence of diffusivity on density at low temperatures, with well-defined maxima and minima that signal anomalous transport behavior. As temperature increases, these anomalies progressively weaken and disappear by 300 K within the explored pressure range, where diffusion becomes a monotonic function of density. While both models capture the qualitative features of anomalous diffusion, the densities and corresponding pressures at which the extrema occur are systematically shifted relative to experimental observations.

At approximately 260 K, experimental measurements place the maximum of the diffusion coefficient near 1500 bar [60], whereas in calculations performed in our simulations, the TIP4P/$\varepsilon$ and OPC3 models predict this maximum at significantly higher pressures, around 3026 bar and 2987 bar, respectively. This systematic shift of the diffusion extrema to higher densities and pressures reflects limitations inherent to rigid non-polarizable water models. In the absence of intramolecular flexibility and electronic polarization, structural rearrangements under compression are governed by a stiffer effective equation of state, requiring higher pressures to access configurations that promote enhanced molecular mobility. This behavior is consistent with the reduced compressibility typically observed in rigid non-polarizable models [2]. As a result, although both models correctly reproduce the qualitative hierarchy of diffusion anomalies, the corresponding state points are displaced relative to experimental observations.

**Fig. 7:**
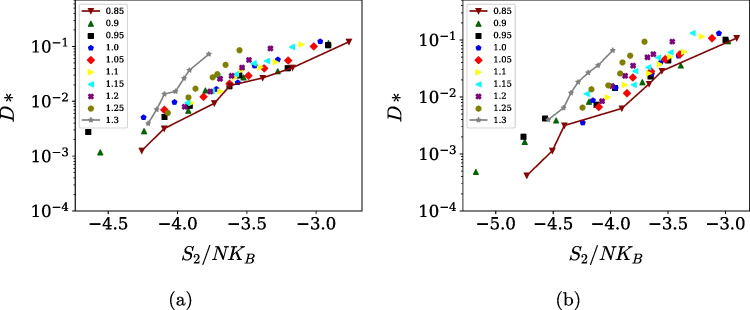
Reduced diffusivity $D^*$ as a function of excess entropy for (**a**) OPC3 and (**b**) TIP4P/$\varepsilon$ models at different temperatures. The reduced diffusivity is defined as $D^* = D \rho ^{1/3}(m/k_B T)^{1/2}$. Symbols correspond to distinct densities, and lines connect state points at fixed temperature from the lowest to the highest density, serving as a guide to the eye

The coupling between structure and dynamics was then examined through the excess entropy derived from pair correlation functions as shown in Fig. 6a for the OPC3 model and in Fig. 6b for the TIP4P/$\varepsilon$ model. Regions where excess entropy exhibits anomalous behavior closely coincide with those where diffusion anomalies are observed, particularly at low temperatures. As temperature increases, the magnitude of both entropy and diffusion anomalies decreases and vanishes by 300 K in the investigated state points, indicating a crossover to a regime characterized by more conventional liquid behavior. This correspondence is consistent with the interpretation that dynamic anomalies in water are rooted in structural rearrangements of the hydrogen-bond network, which are directly reflected in the pair correlation entropy.

Finally, the relationship between excess entropy and transport was examined within the Rosenfeld scaling framework as shown in Fig. 7a for the OPC3 model and in Fig. 7b for the TIP4P/$\varepsilon$ model. Rosenfeld scaling for the reduced diffusivity utilizes macroscopic reduction derived from elementary kinetic theory, which can be defined as $D^* = D \rho ^{1/3}(m/k_B T)^{1/2}$. Along individual densities, both models display an approximately linear relationship between reduced diffusivity and excess entropy, consistent with Rosenfeld’s scaling [3, 61]. Deviations from linearity become more pronounced at lower temperatures, where cooperative dynamics and structural heterogeneity are enhanced [62]. These deviations are expected in regimes where local structural motifs compete and where higher-order correlations become increasingly relevant. Overall, the results show that, despite their simplicity, rigid three-site and four-site water models are capable of capturing not only individual anomalies but also the deeper thermodynamic–dynamic correlations that underpin anomalous behavior in liquid water.

## Conclusions

In this paper, we presented a comparative study of the rigid non-polarizable OPC3 and TIP4P/$\varepsilon$ water models, using structural, thermodynamic, and dynamic anomalies as the primary framework for evaluation as they represent the hierarchy of the anomalous behavior of water [45, 46]. The two force fields reproduce the essential features of liquid water, including the temperature dependence of structural correlations and the qualitative hierarchy of water-like anomalies.

Clear differences arise from model architecture. The four-site TIP4P/$\varepsilon$ model provides a more accurate description of short-range structure and density-related anomalies, including an excellent reproduction of the temperature of maximum density, whereas the three-site OPC3 model yields softer intermediate-range correlations and competitive performance for second-shell structure. Dynamic anomalies are captured by both models but are systematically shifted to higher densities and pressures relative to the experiments, reflecting constraints imposed by the rigid molecular framework, with secondary differences arising from the number of interaction sites.

The analysis of excess entropy reveals a direct connection between structural organization and molecular mobility and supports the approximate validity of Rosenfeld scaling along isochores. Overall, these results show that modern rigid water models remain effective for probing anomalous behavior and structure–dynamics relationships, while clarifying the distinct compromises introduced by three-site and four-site parameterizations, thereby providing guidance for the rational selection of water models in large-scale molecular simulations.

There are studies in the literature comparing structural, thermodynamic, and dynamic properties between rigid and flexible water models. Valle et al. [25] compared the rigid TIP4P/2005 model with the flexible SPC/Fw model and found that the rigid model provides a more accurate description of the density anomaly, while both models show comparable diffusion coefficients and similar O–O radial distribution functions. Differences emerge at the level of O–H correlations, reflecting the enhanced ability of flexible models to rearrange the hydrogen-bond network.

Earlier works by Tironi et al. [63] and Wallqvist and Teleman [64] also reported that flexibility has a limited impact on structural and dynamical properties, with only minor differences in radial distribution functions and transport coefficients. These results suggest that the main anomalies of water are largely preserved across rigid and flexible models, although quantitative shifts may occur depending on the parametrization.

In this context, our findings indicate that the differences observed between OPC3 and TIP4P/$\varepsilon$ are primarily associated with their parameterization strategies rather than with the presence or absence of intramolecular flexibility. Recent studies on core-softened dimeric systems have shown that internal flexibility can significantly modify both structural organization and the hierarchy of thermodynamic and dynamic anomalies, acting as a key control parameter in anomalous fluids [65, 66]. Together, these results highlight that both parameterization and internal degrees of freedom play complementary roles in shaping the anomalous behavior of complex fluids.

## Supplementary Information

Below is the link to the electronic supplementary material.

Supplementary file 1 (pdf 165 KB)

## Data Availability

Data sets generated during the current study are available from the corresponding author on request. All LAMMPS input files used to generate the results reported in this work are available in a public repository at https://github.com/Bordin-Lab/water-comparison.
