# Supplementary material for: Molecular dynamics study of water anomalies: a comparison of OPC3 and TIP4P/ε models
Source: J Mol Model. 2026 May 15;32(6):190. doi: 10.1007/s00894-026-06748-x (PMC13179192; doi:10.1007/s00894-026-06748-x)
Supplement: Supplementary file 1 — (pdf 165 KB) [file 894_2026_6748_MOESM1_ESM.pdf]

Supplementary Material

Molecular Dynamics Study of Water Anomalies: A comparison of OPC3 and TIP4P/ε models

Vanderson dos S. Nascimento, Rogelma M. S. Ferreira, Rogelma M. S. Ferreira, José Rafael Bordin

MSDs

Figures S1 and S2 show the MSD for the calculation of the diffusion coefficient for the OPC3 and TIP4P/ε models, respectively. Each MSD was divided into four segments; a linear fit was performed on each segment, and the average was calculated to achieve greater precision in the results.

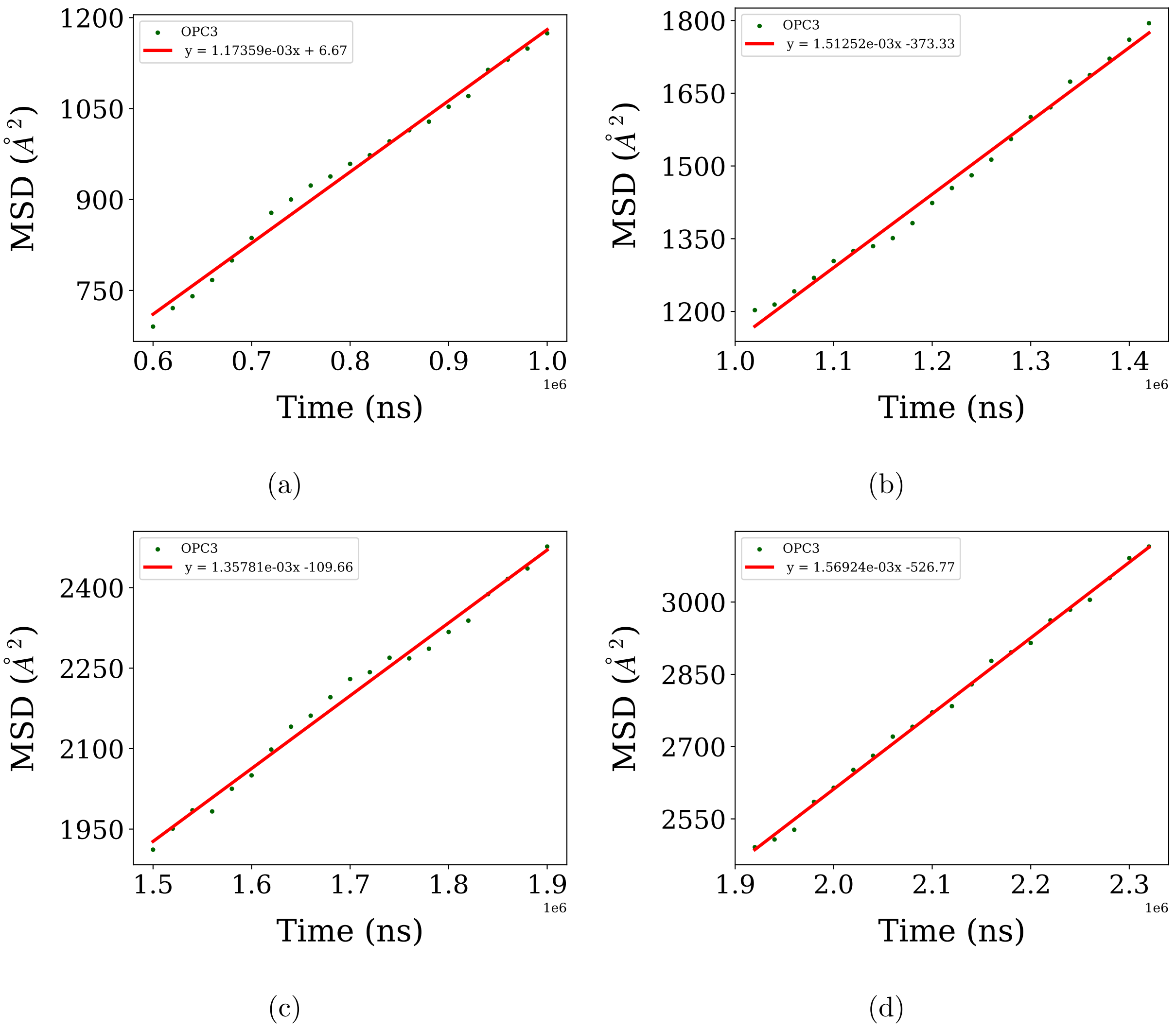

FIG. S1: MSD of water at a temperature of 300 K for the OPC3 model. Each MSD was divided into four segments; a linear fit was performed on each segment, and the average was calculated to achieve greater precision in the results.

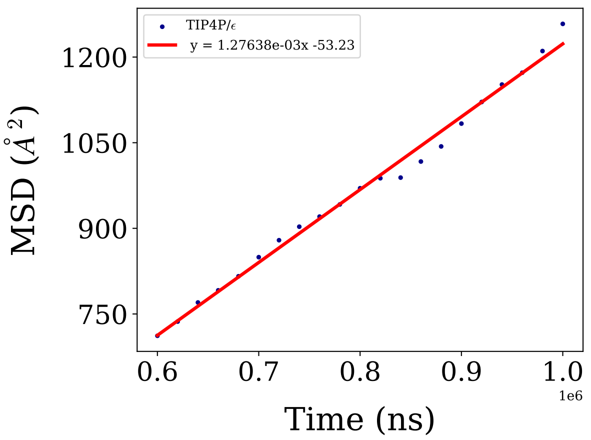

(a)

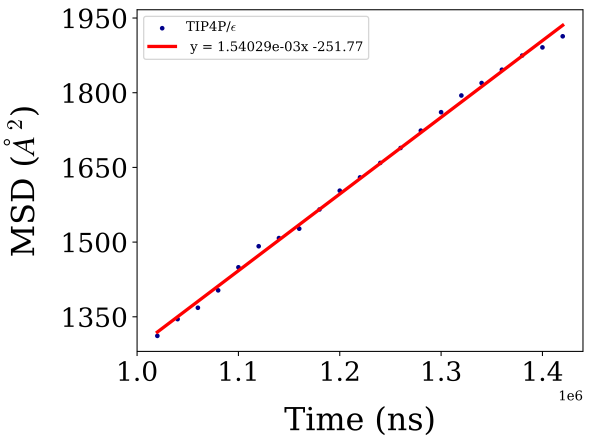

(b)

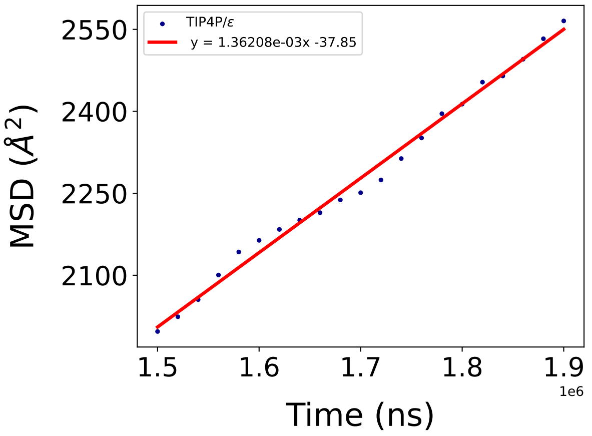

(c)

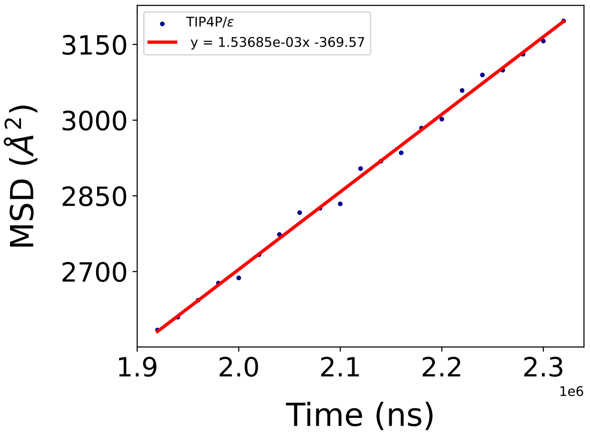

(d)

FIG. S2: MSD of water at a temperature of 300 K for the TIP4P/ε model. Each MSD was divided into four segments; a linear fit was performed on each segment, and the average was calculated to achieve greater precision in the results.
